# The Vaccination Bill

**Published:** 1898-08-06

**Authors:** 


### The Hospital

A Journal of

The Medical Sciences and Hospital Administration.

Vol. XXIV.—No. 619.] Edited by Sir Henry Burdett, K.C.B., and Solomon C. Smith, M.D., M.R.C.P. [Aug. 6, 1898.

### THE VACCINATION BILL.

Unless the British public should once more be furnished with reason to be thankful for the possession of a House of Lords, the very remarkable piece of legislation commonly known as the Vaccination Bill, and which might more appropriately be described as a Bill for the promotion of small-pox, will find its way to the roll of Parliament at the close of the present session. We think it was Charles Lamb who, when reproved by an official superior for always arriving late at the India Office, replied that he made the matter straight by always going away early; and it must be by a somewhat similar process of reasoning that Ministers, and some of their supporters in the House of Commons, among whom we regret to have to include Sir Walter Foster, have persuaded themselves, and have attempted to persuade others, that the abolition of compulsion will promote the practice of vaccination. With a few insignificant exceptions, all educated and intelligent persons will be careful to have their children vaccinated and revaccinated, after the Bill has passed, precisely as they would have done aforetime. But educated and intelligent persons, in any proper sense of either word, must always form a comparatively small proportion of a great people; and the national efficacy of vaccination depends upon the action of the numerical majority. The wife of the average artisan, or factory hand, or agricultural labourer, has not, and cannot have, any conception of what is meant by the prevalence of small-pox in a country or community, and cannot understand the magnitude of the danger from which it is proposed that her children should be protected. She is by no means wanting either in maternal instinct or in maternal affection, and she would do for her family anything which she was convinced to be right and necessary. But she is human, she is hard-worked, and she dislikes to take trouble which she thinks might be avoided. She knows that a vaccinated infant is likely to be restless and fretful for a day or two; and she has heard, as who has not, accounts of the dreadful consequences which have followed the operation. Like all ignorant people, she is credulous, and the indulgence of her credulity at least makes for her immediate ease. In this combination of circumstances she will be visited, soon after the birth of her baby, by a fluent person in shabby black, who will probably be paid by commission upon his successes, and who will bring her a form of application to a bench of magistrates for exemption from the penalty which would otherwise attach to neglect of vaccination. The fluent person will enlarge upon the evils and inconveniences of the operation, will point out how easily it may be performed at any future time if small-pox should make its appearance in the locality; will tell her how Mrs. Jones (who will always live at least ten miles away) has lately lost her child in consequence of vaccination, and will say that, if the process is to be undergone at all, it certainly should not be until a later period of life. The only antagonistic influence will be that of the local doctor, who will have but scanty time to spend in argument, who will possibly content himself with telling the mother not to be a fool, and who, she will be assured by the anti-vaccination agent, has a large pecuniary stake in the question. It requires no particular power of prophecy in order to foretell the result. Vaccination, among the poorer classes, will be suffered to fall into almost absolute desuetude, and the country before long will be visited by epidemics of small-pox such as those which were the scourges of the eighteenth century.

In view of this prospect, and in preparation for what must inevitably occur, it will be the duty of local sanitary authorities carefully to watch the returns of vaccination in their several districts, and, as these fall off, to make preparation for the consequences by an increased provision of isolation hospitals. These, as the recent experience of Gloucester has fully shown, do not at present exist of sufficient magnitude, or in sufficient number, to meet the requirements of the near future. It is well known that the local authorities of Leicester have hitherto succeeded in avoiding an epidemic by the manner in which they have gone beyond their legal powers, and have practically enforced imprisonment upon sufferers from small-pox, and upon all who had been brought into communication with them; and the same system will have to be adopted throughout England if the worst consequences of the surrender of the Government to faddists, consequences which cannot be altogether avoided, are at least to be minimised. The anti-vaccinators, and unfortunately not the anti-vaccinators alone, must be prepared to pay, both in money and by a loss of liberty when small-pox actually arrives, for their liberty to do evil while it is still only on the horizon. In the meantime, the medical profession is free from blame. It has warned the public with no uncertain voice, and, if the public does not chose to profit by the warning, it must at least be prepared to accept the consequences.

